# Net1 and Myeov: computationally identified mediators of gastric cancer

**DOI:** 10.1038/sj.bjc.6603054

**Published:** 2006-03-21

**Authors:** J Leyden, D Murray, A Moss, M Arumuguma, E Doyle, G McEntee, C O'Keane, P Doran, P MacMathuna

**Affiliations:** 1 Gastrointestinal Unit, Mater Misericordiae University Hospital, University College Dublin, Ireland; 2 Genome Resource Unit, Department of Medicine and Therapeutics, Mater Misericordiae University Hospital, University College Dublin, Dublin 7, Ireland; 3 Department of Surgery, Mater Misericordiae University Hospital, University College Dublin, Ireland; 4 Department of Pathology, Mater Misericordiae University Hospital, University College Dublin, Ireland

**Keywords:** Net1, Myeov, gastric cancer, DDD

## Abstract

Gastric adenocarcinoma (GA) is a significant cause of mortality worldwide. The molecular mechanisms of GA remain poorly characterised. Our aim was to characterise the functional activity of the computationally identified genes, NET 1 and MYEOV in GA. Digital Differential Display was used to identify genes altered expression in GA-derived EST libraries. mRNA levels of a subset of genes were quantitated by qPCR in a panel of cell lines and tumour tissue. The effect of pro- and anti-inflammatory stimuli on gene expression was investigated. Cell proliferation and invasion were measured using in an *in-vitro* GA model following inhibition of expression using siRNA. In all, 23 genes not previously reported in association with GA were identified. Two genes, Net1 and Myeov, were selected for further analysis and increased expression was detected in GA tissue compared to paired normal tissue using quantitative PCR. siRNA-mediated downregulation of Net1 and Myeov resulted in decreased proliferation and invasion of gastric cancer cells *in vitro*. These functional studies highlight a putative role for NET1 and Myeov in the development and progression of gastric cancer. These genes may provide important targets for intervention in GA, evidenced by their role in promoting invasion and proliferation, key phenotypic hallmarks of cancer cells.

Gastric cancer is the second most common cause of cancer-related mortality worldwide, with highest incidence in Japan and China (Parkin *et al*, 2001). While the incidence of gastric cancer has been falling in recent decades, the overall 5-year survival for gastric cancer remains poor, with European rates varying from 12 to 28% (Newnham *et al*, 2003). This poor survival, despite advances in both diagnostic and therapeutic modalities, is primarily due to the advanced stage of disease at presentation. Adenocarcinomas represent the majority of gastric cancers and histologically, can be classified into two distinct types – intestinal and diffuse (Faivre *et al*, 1998). Intestinal type gastric adenocarcinoma (GA) is the most common and is believed to arise as a result of chronic inflammation inducing intestinal metaplasia, subsequent dysplasia and finally cancer (Lauren, 1965). Diffuse-type GA is characterised by both solitary or poorly organised clusters of mucin-rich cells and a diffuse infiltrating growth pattern. Unlike intestinal type GA, no identifiable precancerous lesions occur. Several environmental factors have been linked to the development of GA, the most significant being the presence of chronic *Helicobacter Pylori* infection (Correa, 1992). While the molecular events underlying the pathogenesis of GA have not been fully elucidated, mutations in, and altered expression of several genes involved in several important cellular processes have been identified, such as the oncogenes bcl-2, *β*-Catenin, c-erbB-2, cyclinE, K-ras, K-sam, c-Met; the tumour suppressor genes APC, DCC, p53, E-Cadherin, p16, p53; and growth factors such as VEGF and EGF (Ming, 1998). Furthermore, high-level microsatellite instability, as a result of alteration in DNA mismatch repair genes, is seen in approximately 15% of diffuse and familial gastric cancer (El-Omar *et al*, 2000; Engel *et al*, 2003; Nardone, 2003).

Recent advances in the field of molecular biology, genomics and bioinformatics have ushered in a new era in cancer research. One can now study global gene expression patterns in specific tissues or cell types. This approach can be used to identify novel cancer markers, oncogenes, tumour suppressor genes, and potential therapeutic targets. Digital Differential Display (DDD) is a web-based bioinformatics tool for generating differential gene expression profiles between cDNA tissue libraries (http://www.ncbi.nlm.nih.gov/UniGene/ddd.cgi). Using the EST profiles of normal and disease cDNA libraries represented in the NCBI UniGene database, DDD compares the number of times that ESTs from different libraries, or pools of libraries, are assigned to a specific UniGene cluster (Scheurle *et al*, 2000; Dennis *et al*, 2002; Pontius *et al*, 2003).

Herein, we describe the expression and functional characterisation of the DDD identified gastric cancer-associated genes Net 1 and Myeov. We verified the expression of these genes in an *in-vitro* GA model and a panel of *ex-vivo* GA and adjacent normal tissue using real-time PCR. Having determined the changes in expression of these genes in GA, their functional importance was assessed using a gene knockdown-based approach. Specifically, siRNA duplexes were used to knockdown mRNA expression of Net1 and Myeov. This approach induced alterations in the cellular phenotype as evidenced by perturbed cell invasion and proliferation, key endpoints in the molecular phenotype of GA.

## MATERIALS AND METHODS

### Cell culture and cell treatments

An *in vitro* model of gastric cancer was established using three GA cell lines: AGS (ECACC, UK), Kato III (ECACC, UK) and 23132/87 (DSMZ, Germany). The AGS and 23132/87 cell lines were established from primary tumours while the KatoIII was derived from metastatic tissue (Sekiguchi *et al*, 1978; Barranco *et al*, 1983; Vollmers *et al*, 1993). AGS cells were cultured in Hams F12 medium containing 10% fetal bovine serum (FBS). KatoIII cells were grown in RPMI 1640 containing 20% FBS and 23132/87 cells were cultured in RPMI 1640 medium containing 10% FBS. All medium was supplemented with 2 mM L-glutamine, 1 U ml^−1^ penicillin and 1 *μ*g ml^−1^ streptomycin. All cells were incubated in a humid 5% CO_2_ atmosphere at 37°C. To investigate the effect of pro- and anti-inflammatory stimuli on gene expression, AGS cells were treated separately with 0, 0.3, 1, 3 and 10 ng ml^−1^ interleukin-1*β*, TNF-*α* and dexamethasone for 4 h under the growth conditions described above. AGS cells were also treated with or without 10 ng ml^−1^ of each treatment for 0, 2, 4, 6 and 8 h. Total RNA was obtained from normal human alveolar epithelial cells (AEC) (Promocell).

### Human tissue

With informed patient consent according to a protocol approved by the local ethics committee, representative samples of GA and adjacent normal gastric mucosa were collected at endoscopy and surgery and immediately stored in an RNAse inhibitor (RNAlater, Sigma-Aldrich, Ireland) at −20°C. Details of the tumour histology, stage, patient age and gender were noted. In addition to these samples total RNA derived from both GA tissue and matched adjacent normal mucosa was obtained (Ambion, UK; Biochain Institute Inc, Hayward, CA, USA; and Stratagene Europe, Amsterdam, The Netherlands). Details of all tissue used in this study are listed in Table 1.

### RNA extraction and PCR

TRIzol™ (Sigma-Aldrich, Ireland) was used to extract RNA as previously described (Murray *et al*, 2004). For extraction from tissue, 1 ml TRIzol™ was added and the tissue was homogenised at 15 000 r.p.m. using a POLYTRON PT 2100 rotor-stator (Kinematica Inc, Newark, NJ, USA). For extraction from cells in culture, cells were washed in PBS and 1 ml of TRIzol™ was added to cells and left for 10 min at room temperature with occasional shaking. A measure of 2 *μ*g of total RNA was treated with DNAse I and reverse transcribed using random hexamers and SuperScript II reverse transcriptase (Invitrogen Ltd, UK). Primers were designed using Primer3 software (http://frodo.wi.mit.edu/cgi-bin/primer3/primer3_www.cgi) and synthesised by Sigma-Aldrich, Ireland. The sequences of primers used for PCR were *β*-actin forward: 5′-GTC ACC TTC ACC GTT CCA G-3′, reverse: 5′-CTC TTC CAG CCT TCC TTC CT-3′, Net1 forward: 5′-CTG TTC ACC TCG GGA CAT TT-3′, reverse: 5′-TGG AGC TGT CAG ACG TTT TG-3′, Myeov forward: 5′-GGG CTC AGT GAA GAG TCT GG-3′, reverse: 5′-CACACC ACG GAG ACA ATG AC-3′, TNFa forward: 5′-TGG TGT GGG TGA GGA GTA CA-3′, TNFa reverse: 5′-AGC CCA TGT TGT AGC AAA CA-3′, MKP1 forward: 5′-TCC TGC CCT TTC TGT ACC TG-3′, MKP1 reverse: 5′-ATG AAG TCA ATG GCC TCG TT-3′. polymerase chain reaction was carried out in a 50 *μ*l mix containing 0.5 *μ*l of Taq polymerase (Invitrogen, UK) and 1 *μ*l of cDNA. Polymerase chain reaction products were run on a 2% agarose gel with a parallel 100 bp DNA ladder (Promega, UK). Real-time PCR was carried out according to the manufacturers' instructions using the LightCycler RNA SYBR Green 1 Amplification Kit (Roche Applied Science) together with the Light Cycler instrument. All measurements were independently repeated three times. The maximum concentration of total RNA template used was 0.5 *μ*g *μ*l^−1^. The following components were added to 1 *μ*l of total RNA in a 20 *μ*l capillary tube: 10.2 *μ*l PCR-grade H_2_O, 4 *μ*l SYBR Green 1 reaction mix, 2.4 *μ*l 25 mM MgCl_2_, 0.4 *μ*l RT–PCR enzyme mix and 1 *μ*l each of forward and reverse primers. The reaction conditions were as follows: reverse transcription: 55°C for 10 min followed by denaturation: 95°C for 30 s. Forty-five PCR cycles were then run at denaturation: 95°C for 10 s; annealing: (*β*-actin: 54°C, Net1: 52°C, Myeov 56°C) for 10 s and extension: 72°C for 13 s. Data analysis using the delta Ct method was performed using the LightCycler version 4.0 software. *β*-actin expression levels were used to normalise Net1 and Myeov expression.

### Gene silencing by RNA interference

Two siRNA duplexes were designed and synthesised for silencing each of the genes Net1 and Myeov (Qiagen Inc. CA, USA). The duplexes had the following sequences: Net1(1) sense, 5′-GGA GGA UGC UAU AUU GAU A-3′; Net1(1) antisense, 5′-UAU CAA UAU AGC AUC CUC C-3′; Net1(2) sense, 5′-GGU GUG GAU UGA UUG GAA A-3′; Net1(2) antisense 5′-UUU CCA AUC AAU CCA CAC C-3′; Myeov(1) sense, 5′-GGA UGU AAG UUA UCA ACU A-3′; Myeov(1) antisense, 5′-UAG UUG AUA ACU UAC AUC C-3′; Myeov(2) sense, 5′-CCA UGA GGU AGC UAC UAA A-3′ and Myeov(2) antisense, 5′-UUU AGU AGC UAC CUC AUG G-3′. A chemically synthesised non-silencing siRNA duplex with the following sequence; sense, 5′-UUC UCC GAA CGU GUC ACG U-3′; antisense, 5′-ACG UGA CAC GUU CGG AGA A-3′ that had no known homology with any mammalian gene was used to control for nonspecific silencing events. A total of 6 × 10^4^ AGS cells were added to each well of a 24-well plate in 500 *μ*l growth media and incubated under the standard conditions of 37°C and 5% CO_2_ in a humid incubator for 24 h. A volume of 98 *μ*l growth medium was mixed with 0.5 *μ*g siRNA (or a combination of 0.25 *μ*g of each) and 3 *μ*l RNAifect (Qiagen). Following incubation, media was removed from the cells and this mix was added dropwise. A volume of 300 *μ*l growth medium was added and the cells were incubated for 48 h under standard conditions before either being assayed as described below of before RNA was extracted as described.

### Cell proliferation assay

A colorimetric MTS assay (Promega) was used to assess the effect of Net1 and Myeov knockdown on AGS cell proliferation. Following siRNA knockdown, 5 × 10^4^ of either control or siRNA-treated cells were added, in triplicate, to each well of a 96-well micro-plate. The total volume was adjusted to 100 *μ*l with growth medium and 20 *μ*l of MTS reagent was added to each well. The micro-plate was incubated for 2 h at 37°C and 5% CO_2_ and absorbance at 492 nm was read using a Rosys Anthos 2010 plate reader (http://www.promega.com/tbs/tb169/tb169.pdf).

### *In vitro* invasion assay

Biocoat Matrigel invasion chambers (BD Biosciences, USA) were used to investigate the effect of siRNA-mediated Myeov and Net1 gene silencing on the *in vitro* invasiveness of the AGS gastric cell line as previously described (Murray *et al*, 2004). Briefly, 5 × 10^4^ AGS cells were seeded into the upper chamber of the 24-well invasion chamber in medium containing 1% serum. A volume of 750 *μ*l of medium containing 20% FBS was added to the outer chambers to act as a chemoattractant for the cells. The plates were then incubated for 48 h in a 5% CO_2_ humidified 37°C incubator. Following incubation cells that had invaded through the membrane were fixed and stained before the membrane was removed and mounted on a slide for microscopic assesment. Invasive cells were visualised at × 40 magnification and the number of cells in five random fields were counted and an average calculated.

### Statistical methods

A student's *t*-test was used to compare the expression profiles, based on RT–PCR mRNA levels, of selected genes in matched GA and normal tissues, and in the 3 GA cell lines. A *P*-value of <0.05 was taken to indicate significant alterations.

## RESULTS

### Differential expression of Net1 and Myeov in gastric cancer cell lines and human tissue

Digital differential display (DDD) was used to compare 185 pooled normal and 201 pooled adult cancer tissue EST libraries with 11 pooled gastric cancer tissue libraries, details of which are listed in Table 2. All gastric cancer tissue libraries analysed were derived from primary tumours. This comparison identified 23 genes whose expression was altered in gastric cancer-derived material (Table 3). Digital differential display was used to calculate the fold difference in the representation of these genes between libraries. Two genes (Net1 and Myeov) not previously studied in gastric cancer were selected for further investigation using an *in vitro* GA model and a panel of matched *ex vivo* GA tissue and adjacent normal tissue. Our group has shown elevated expression of Myeov, a putative oncogene, initially described in Multiple Myeloma (Janssen *et al*, 2000), in colorectal adenomas and cancer compared to normal colonic mucosa (Moss *et al*, 2004). Net1 is a member of the guanine nucleotide exchange factor (GEF) family which are involved, through their regulation of RhoA activity, in a range of biological processes including cell proliferation, apoptosis, differentiation and cytoskeletal reorganisation (Rossman *et al*, 2005).

**Figure 1:**
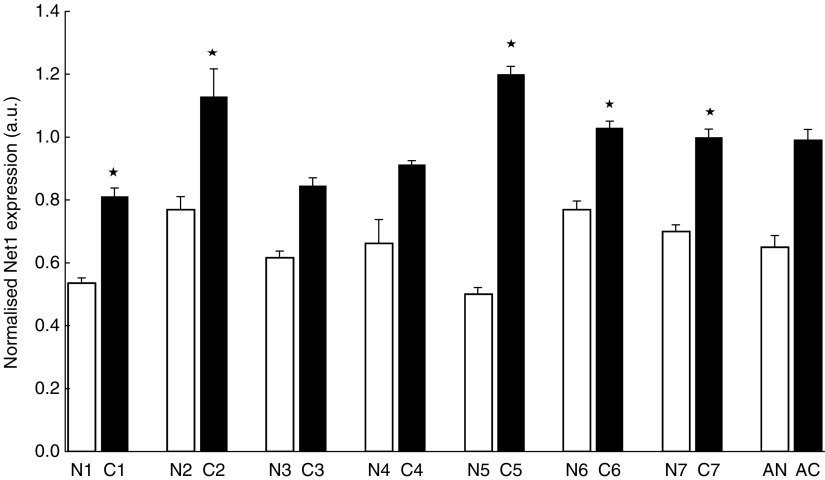
Net1 expression in paired gastric normal and tumour tissue. Real-time PCR was used to investigate the levels of Net1 expression in matched gastric cancer (C) and normal (N) tissue. Each tissue specimen was analysed in triplicate for mRNA levels. *β*-actin expression was used to normalise the data. ^*^(*P*<0.05). Average Net1 expression in cancer (AC) and normal (AN) tissue is displayed.

**Figure 2:**
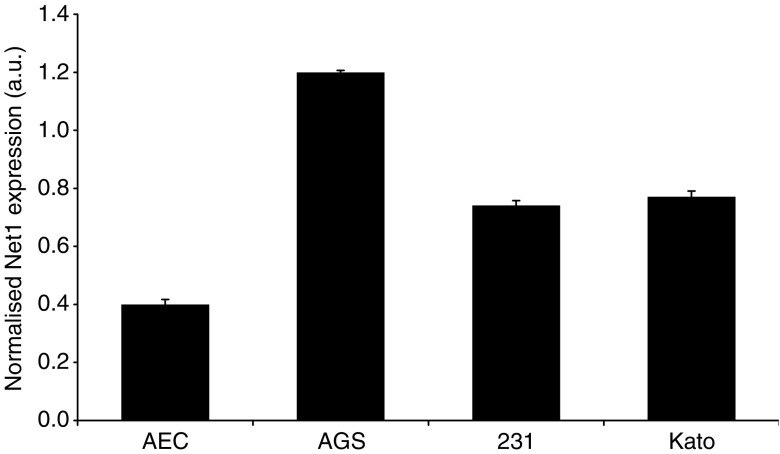
Net1 expression in normal epithelial and gastric cancer cell lines. Real-time PCR was used to determine the relative expression levels of Net1 mRNA in normal alveolar epithelial cells (AEC) and in three gastric cancer cell lines (AGS, 23132/87 (231) and KatoIII (Kato). Each sample was analysed in triplicate for mRNA levels. *β*-actin expression was used to normalise the data.

**Figure 3:**
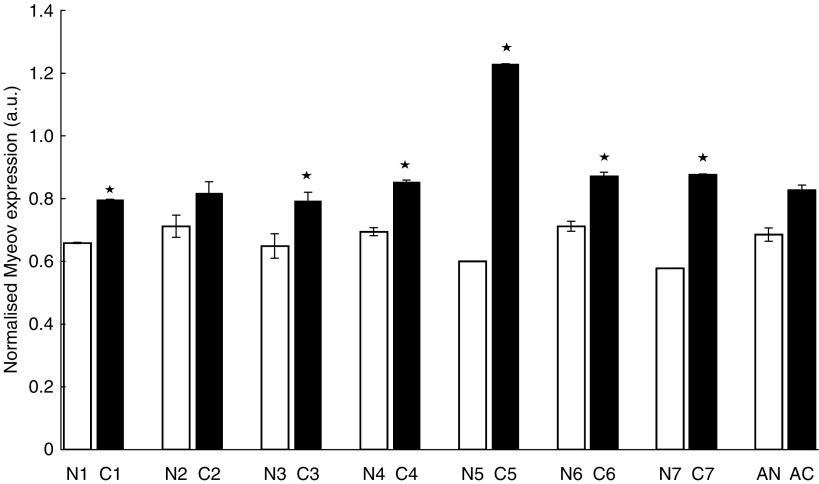
Myeov expression in paired gastric normal and tumour tissue. Real-time PCR was used to investigate the levels of Myeov expression in paired gastric cancer (C) and normal (N) tissue. Each tissue specimen was analysed in triplicate for mRNA levels. *β*-actin expression levels were used to normalise the data. *β*-actin expression was used to normalise all data. ^*^(*P*<0.05). Average Myeov expression in cancer (AC) and normal (AN) tissue is displayed.

**Figure 4:**
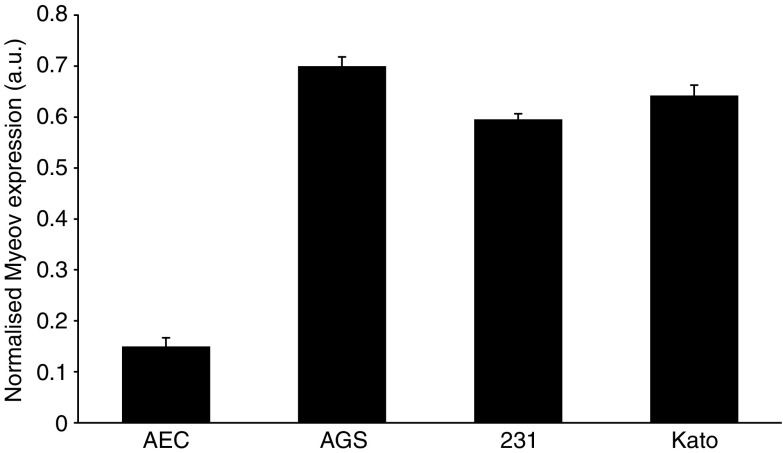
Myeov expression in normal epithelial and gastric cancer cell lines. Real-time PCR was used to determine the relative expression levels of Myeov mRNA in normal alveolar epithelial cells (AEC) and in three gastric cancer cell lines (AGS, 23132/87 (231) and KatoIII (Kato). Each sample was analysed in triplicate and *β*-actin expression was used to normalise the data.

To investigate the expression of Net1 and Myeov in gastric cancer, real-time PCR was used to determine mRNA levels in tissue and cells lines. Using *β*-actin mRNA expression to normalise, quantitative PCR confirmed elevated levels of Net1 expression in all cancer tissue specimens studied, in comparison with adjacent normal tissue (Figure 1). Net1 expression was significantly increased by 34, 32, 58, 25 and 30% in five of the seven paired tumour tissue samples studied (*P*<0.05). This confirmed the enhanced expression identified using DDD. Expression of Net1 was shown in three separate gastric cancer cell lines, namely AGS, 23132 and KatoIII (Figure 2). Net1 expression in a non-cancerous airway alveolar epithelial cell line (AEC) was less than that in the gastric cancer cell lines. Similarly, increased Myeov expression in gastric cancer tissue in comparison with normal tissue from the same patient was confirmed using real-time PCR (Figure 3). All gastric cancer tissue studied, expressed higher levels of Myeov mRNA than adjacent normal tissue. Myeov expression was significantly increased by 17, 18, 19, 51, 18 and 34% in six of the seven tumour tissue samples studied in comparison with adjacent normal tissue (*P*<0.05). The increased levels of Myeov in gastric cancer tissue confirmed the observations made using DDD. Myeov expression was also detected in all gastric cancer cell lines studied (Figure 4). The expression of Myeov in AEC cells was not as high as its expression in gastric cancer cell lines.

### The expression of Net1 and Myeov in response to inflammatory stimuli

**Figure 5:**
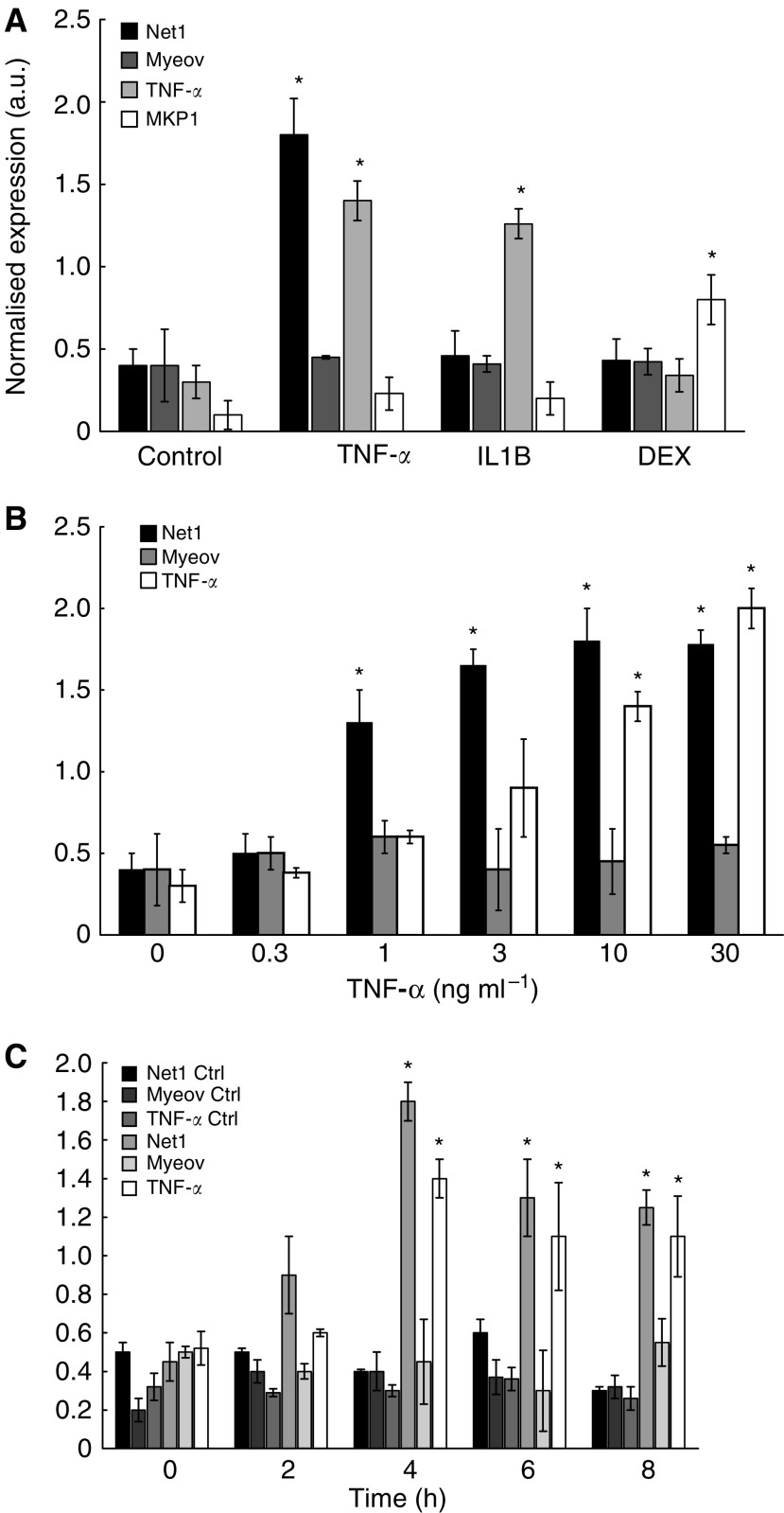
The effect of pro- and anti-inflammatory stimuli on Net1 and Myeov expression in gastric cancer. (**A**) The effect of 10 ng ml^−1^ interleukin-1*β* (IL-1B), TNF-alpha (TNFa) and Dexamethasone (DEX) on Net1 and Myeov expression in AGS gastric cancer cells was monitored using real-time PCR. Cells were treated for 4 h. Enhanced TNFa expression was used as a positive response to TNFa and IL1B treatment (Gallagher *et al*, 2003). Enhanced MKP1 expression was used a positive response to DEX treatment (Lasa *et al*, 2002). Apart from the positive controls, the only trteatment to have a significant effect was TNF*α*, which resulted in a threefold increase in Net1 mRNA expression ^*^(*P*<0.05). Enhanced TNFa-induced Net1 expression resulted in a dose-dependent (**B**) and time-dependent (**C**) manner ^*^(*P*<0.05).

Inflammation is a key process underpinning the progression of GA (Kai *et al*, 2005), thus the putative role of inflammatory mediators in driving Net1 and Myeov expression was further investigated. Cells were treated with specific pro- and anti-inflammatory stimuli to investigate to effect on Net1 and Myeov expression in response to inflammation. Net1 mRNA expression increased in a dose- and time-responsive manner to treatment with TNF*α* (Figure 5). Treatment with 10 ng ml^−1^ TNF*α* resulted in a threefold increase in Net1 expression after 4 h (*P*<0.05). Treatment with IL-1b or dexamethasone did not have a significant effect on Net1 expression (Figure 5). Treatment with either TNF*α*, IL1*β* or dexamethasone did not have a significant effect on Myeov expression (Figure 5). This data suggests that these genes respond in a stimulus-specific manner.

### Effect of Net1 and Myeov knockdown on cellular proliferation

**Figure 6:**
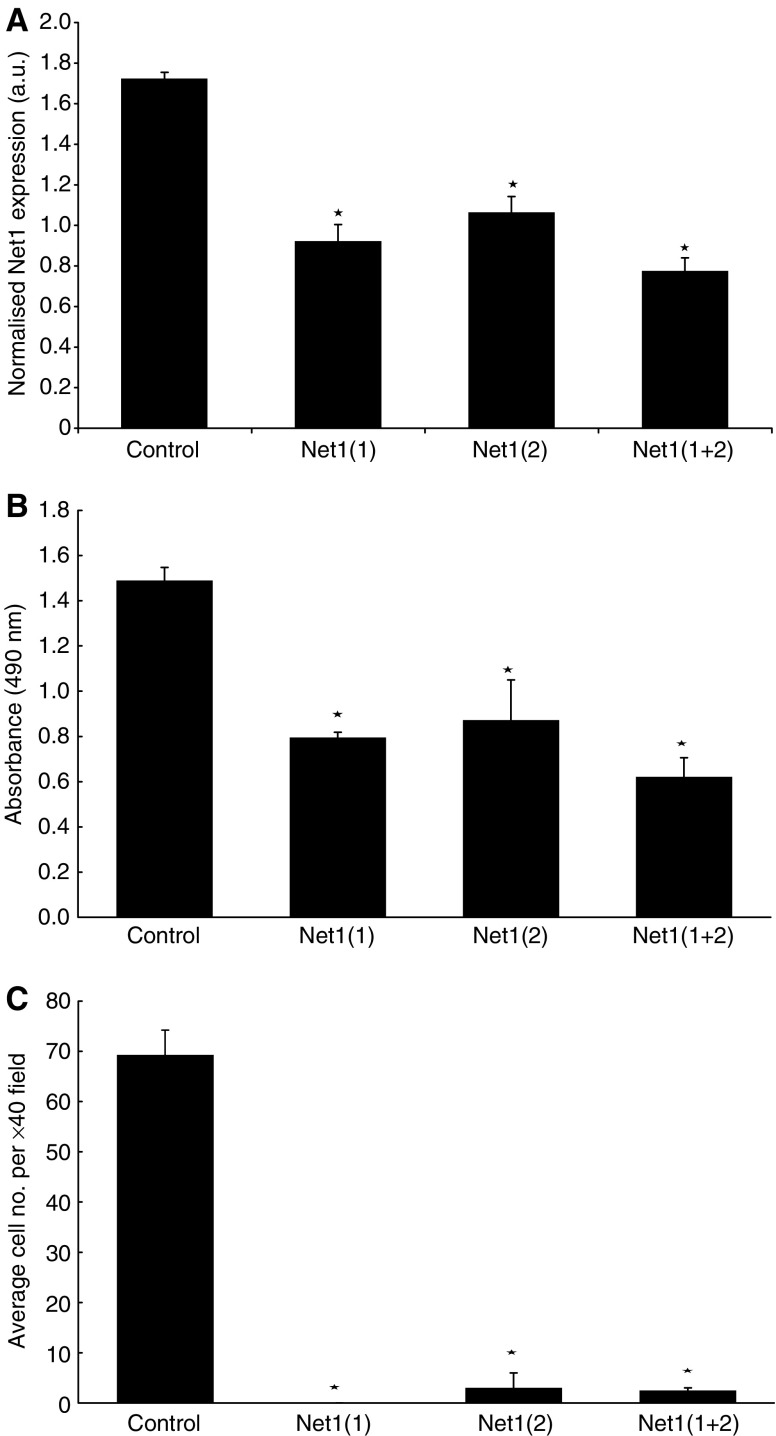
The effect of Net1 knockdown on gastric adenocarcinoma cell proliferation and invasion. (**A**) Real-time PCR was used to confirm the significant siRNA-mediated reduction in Net1 expression. Using two siRNA duplexes; Net1(1) and Net1(2), Net 1 expression was reduced by 49 and 41%, respectively, in comparison with control cells. Using a combination of both siRNA duplexes; Net1(1+2) resulted in 55% decreased Net1 expression (*P*<0.05). (**B**) The proliferation of cells treated with both duplexes was compared with control cells using an MTS assay. siRNA mediated Net1 downregulation resulted in 47 and 41% decrease in cell proliferation. Using both Net1 siRNA duplexes in combination resulted in 58% decreased cell proliferation (*P*<0.05). (**C**) Downregulation of Net1 expression using both siRNA duplexes resulted in 100 and 96% reduction in cell invasion in comparison with control cells. A combination of both siRNA molecules resulted in 96% AGS cell invasion ^*^(*P*<0.05).

**Figure 7:**
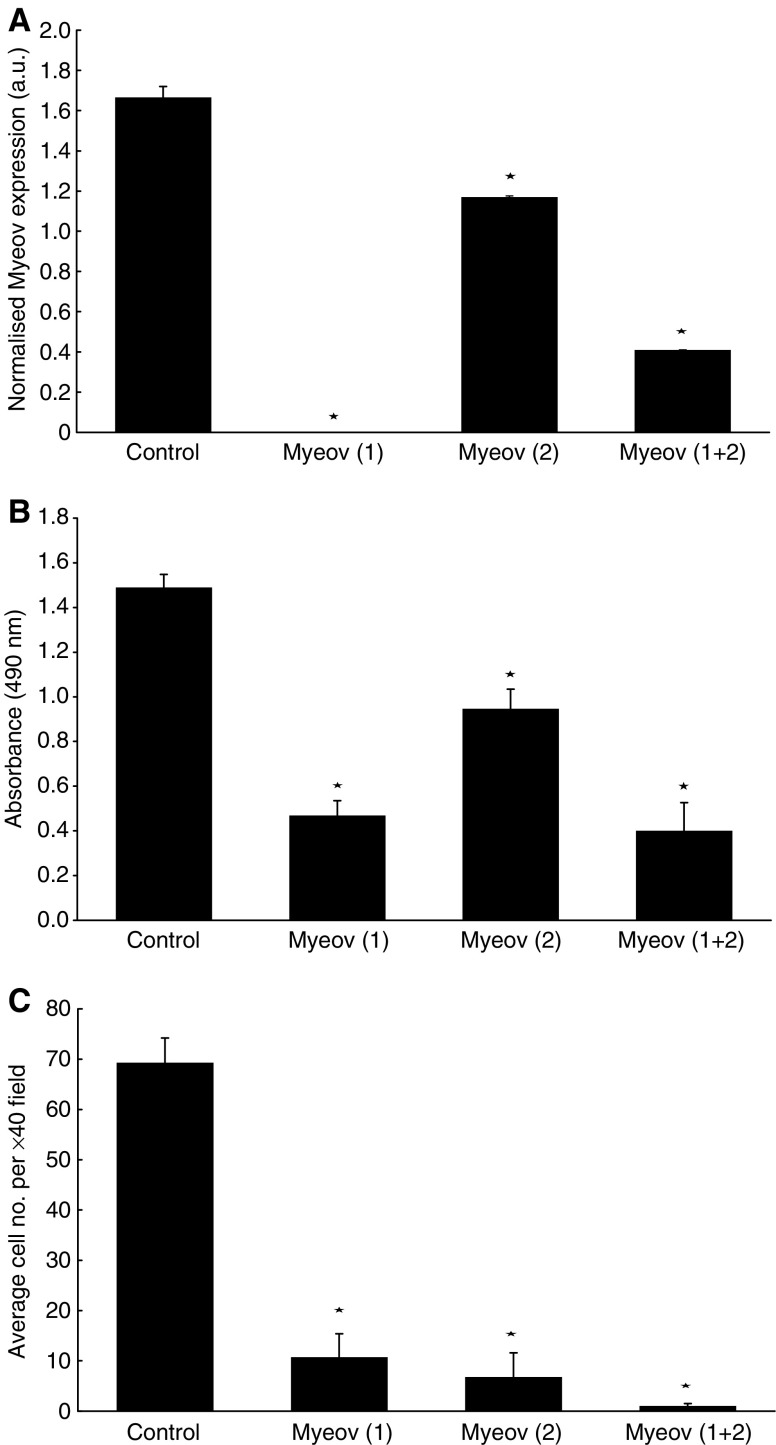
The effect of siRNA-mediated Myeov knockdown on AGS cell proliferation and invasion. (**A**) Confirmation of Myeov downregulation using real-time PCR. Myeov downregulation, using two separate siRNA duplexes, Myeov(1) and Myeov(2), resulted in 100 and 30% decreased expression when compared with control cells. A combination of both Myeov siRNA molecules resulted in 75% decreased Myeov expression (*P*<0.05). (**B**) Using siRNA duplexes, cell proliferation was reduced 68 and 36%, respectively, when compared with control cells. Using both duplexes in combination resulted in 73% decrease in gastric cancer cell proliferation (*P*<0.05). (**C**) Similarly, cell invasion was reduced by 85 and 90% and by 99% using a combination of both siRNA duplexes in comparison with control cells ^*^(*P*<0.05).

Having identified enhanced expression of Net1 and Myeov in GA, the functional consequence of their expression was studied. Specifically, the functional effect of Net1 and Myeov gene knockdown using a silencing RNA approach was determined. Using AGS gastric cancer cells, siRNA was used to suppress gene expression. Two siRNA duplexes were designed to target each transcript and gene silencing was confirmed using real-time PCR. Using two separate siRNA duplexes, Net1 expression was reduced by 49 and 41% (Figure 6A) (*P*<0.05). Combining both duplexes resulted in a 55% reduction in Net1 expression. Cells in which Net1 expression was reduced had significantly lower proliferation rates in compassion with the same cells in which Net1 mRNA was unperturbed (Figure 6B). Using both siRNA duplexes seperataly, cell proliferation was decreased by 47 and 41% in comparison with control cells (*P*<0.05). Cell proliferation was decreased by 58% using both duplexes in combination. Similarly, two siRNA duplexes were used to supress Myeov expression by 100 and 30% (Figure 7A) (*P*<0.05) which in turn led to 68 and 36% reduction in gastric cancer-cell proliferation (Figure 7B) (*P*<0.05). Using both siRNA duplexes in combination resulted in a 75% decrease in Myeov expression (*P*<0.05) and a 73% decrease in AGS cell proliferation (*P*<0.05). These data demonstrate that the enhanced expression of Net1 and Myeov in the setting of GA has a significant effect on gastric epithelial tumour cell biology.

### Effect of Net1 and Myeov knockdown on *in vitro* invasion

To further demonstrate the putative role of Net1 and Myeov in gastric cancer, the effect of RNAi-mediated mRNA downregulation on the *in vitro* invasion of AGS cells was monitored. The Net1-targetted siRNA duplexes resulted in 49 and 41% knockdown in mRNA expression (Figure 6A) and the same duplexes caused 100 and 96% decrease in cell invasion (Figure 6C) (*P*<0.05). Using both siRNA suplexes in combination resulted in a 55% reduction in Net1 mRNA expression and a 96% reduction in *in vitro* cell invasion (*P*<0.05). Reduction in Myeov mRNA by 100 and 30% using two separate RNAi duplexes (Figure 7A) resulted in 85 and 90% decreased cell invasion (Figure 7C) (*P*<0.05). Using both siRNA molecules resulted in a 75% reduction in Myeov expression and a 99% decrease in AGS cell invasion (*P*<0.05).

## DISCUSSION

Gastric adenocarcinoma is a significant global cause of morbidity and mortality, with no satisfactory therapy available. Therefore, the development of novel diagnostic and prognostic markers, coupled with enhanced therapeutic options is a key goal for the cancer research community. This goal can only be realistically achieved by further improving our knowledge of the gastric tumour at a molecular level. Examination of EST libraries from both gastric tumours and normal tissue identified a cohort of genes with differential representation in cancer-derived libraries.

Neuroepithelial transforming gene-1 (Net1) is a guanine nucleotide exchange factor (GEF) that activates Rho family proteins (Symons and Rusk, 2003). The Net1 gene was originally isolated in a tissue culture screen for novel oncogenes in NIH 3T3 fibroblasts (Chan *et al*, 1996; Alberts and Treisman, 1998). GEFs regulate Rho- GTPases, a main branch of the Ras superfamily of small (∼21 kDa) GTPases. Rho proteins, once activated, stimulate signalling in multiple pathways by binding to downstream effector proteins, modulating their activities and thereby regulating a range of cellular processes including cell proliferation, apoptosis, differentiation and cytoskeletal reorganisation. They are also thought to play a role in transformation and metastasis (Yoshioka *et al*, 1999; Etienne-Manneville and Hall, 2002; Jaffe and Hall, 2002; Sahai and Marshall, 2002). Other GEFs with established roles in various malignancies include Bcr (Advani and Pendergast, 2002) Tiam (Malliri *et al*, 2002) and Vav1 (Fernandez-Zapico *et al*, 2005). Owing to the importance of GEFs in normal cellular processes and in malignancies and also because there is no published evidence to support its role in the gastric cancer setting Net1 was chosen for further validation and investigation.

mRNA expression determination, using real-time PCR, confirmed elevated Net1 expression in gastric cancer tissue in comparison with normal tissue (Figure 1). Net1 was further shown to be expressed in three separate gastric cancer cell lines (Data not shown). In this study, Net1 expression was responsive in a dose- and time-dependent manner to the pro-inflammatory cytokine TNF-*α* (Figure 5). There was no significant effect on Net1 expression in response to treatment with IL1*β* or with the anti-inflammatory steroid dexamethasone. These data suggest that in the disease setting, Net1 expression is increased in response to inflammation, a common driving factor of GA. To delineate the functional role of Net1 in gastric cancer, RNAi technology was employed to knockdown expression and the effect on cellular proliferation and invasion was monitored. Net1 is a RhoA-specific GEF (Kawasaki *et al*, 2003). Elevated levels of Rho activity have been shown to downregulate the cell cycle regulator p21/Waf1 thus inducing cell proliferation in Swiss-3T3 cells (Sahai *et al*, 2001). Recently, it has been demonstrated that abolition of RhoA expression in AGS cells resulted in decreased proliferation (Liu *et al*, 2004). In this study a reduction in Net1 expression resulted in a significant decrease in the proliferation of GA cells (Figure 4B). Using two siRNA duplexes that targeted Net1 mRNA, AGS cells with Net1 knockdown showed 47 and 41% decreased proliferation, in comparison with control cells in which Net1 expression was unchanged. Net 1 is a GEF for RhoA, which has been previously shown to promote the cell cycle by inducing cyclin D1 expression via the AP-1 transcription factor (Hill *et al*, 1995; Marinissen *et al*, 2001). Increased cyclin D1 expression has been shown to be associated with uncontrolled gastric cancer cell proliferation (Song *et al*, 2003). Cyclin D1 is a critical regulator of normal progression of cells through G1-S transition via the activity of cyclin D1-dependent regulatory proteins (Shie *et al*, 2000; Yu *et al*, 2000). In addition, in a study utilising a mouse model in which hyperproliferation/hyperplasia of the colon was induced, cyclin D1 protein levels measured in tissue extracts were significantly enhanced (Sellin *et al*, 2001). Furthermore, it has been demonstrated in a clinical analysis that nearly 50% of tissue samples examined from 70 colorectal cancer patients expressed increased levels of cyclin D1 (Utsunomiya *et al*, 2001). These studies suggest that one of the potential mechanisms of tumour growth promotion may include the disregulation of cyclin D1.

The *in vitro* invasion of AGS gastric cancer cells following Net1 knockdown was also assessed. siRNA-mediated Net1 knockdown using two separate duplexes significantly reduced the invasive capacity of these cells by 100 and 96% in comparison with control cells in which the Net1 expression was unaltered (Figure 4C). Invasion is an essential event in the malignancy of gastric cancer and this finding further underpins the role of Net1 in mediating this process.

Given the importance of Rho proteins in the motility of normal cells and their aberrant regulation in transformed cells, it is likely that they are involved in the invasion of tumour cells. RhoA has been shown to induce the AP-1 transcription factor, which is known to regulate matrix metalloproteinase (MMP) expression (Benbow and Brinckerhoff, 1997; Marinissen *et al*, 2004). The roles of MMPs in malignant cancers are well established, where elevated expression occurs in areas of active invasion (Sternlicht and Bergers, 2000). Rho proteins have been shown to mediate MMP-2 activation and enhance cell invasion (Zhuge and Xu, 2001). It is therefore likely that the RhoA-mediated increase in AP-1 activity leads to increased MMP expression thus supporting the motile and invasive phenotype. As well as increased ECM turnover and proteolytic activity, cyoskeletal reorganisation is a crucial aspect of cellular locomotion and its disregulation is often a hallmark of invasive tumour cells. RhoA can regulate the function of the ERM (ezrin, radixin, moesin) family of adaptor proteins (Matsui *et al*, 1998). ERM proteins promote cell motility by functioning as molecular linkers between the plasma membrane and the actin cytoskeleton through their association with the cell adhesion molecule CD44 (Tsukita *et al*, 1994; Bretscher *et al*, 2002; Martin *et al*, 2003). CD44 upregulation has been reported in various invasive tumours and have previously demonstrated that CD44 activation leads to enhanced MMP expression and increased cell invasion in colorectal cancer (Murray *et al*, 2004). RhoA function therefore promotes both cell motility and ECM turnover, thus linking two key metastatic events. It is therefore likely that elevated levels of Net1 in gastric cancers favours tumour proliferation and invasion through RhoA activation. The specific mechanisms of Net1 activation remain to be established and the exact role of Net1 in controlling proliferation and invasion requires further investigation.

Myeov was initially identified as a transforming gene through the application of a NIH/3T3 tumorigenicity assay to DNA from a gastric carcinoma however its role in gastric cancer remains unclear (Janssen *et al*, 1999). Myeov maps to the 11q13 region. This region of chromosome 11 contains several genes, most notably CyclinD1 and ESM1/Cortactin, which are frequently overexpressed, mainly through amplification, in a variety of human cancers (Schuuring, 1995; Ormandy *et al*, 2003). Myeov has been shown to be activated concurrently with CyclinD1 in multiple myeloma, breast cancer and esophageal squamous cell carcinomas (Janssen *et al*, 2002a, 2002b). Sequence analysis predicted Myeov is a 313-amino-acid protein containing no known functional motifs except for an RNP1 motif typical of RNA-binding proteins (Kai *et al*, 2005). Herein we have evaluated the expression and biological significance of Myeov in human gastric cancer to determine its role in the disease process.

Real-time PCR confirmed elevated Myeov mRNA levels in gastric cancer tissue and cell lines in comparison with its expression in normal tissue (Figure 2A). AGS cells were treated with specific pro- and anti-inflammatory cytokines, yet neither had a significant effect on Myeov expression (Figure 5), suggesting that the role of Myeov in GA is independent of inflammation and not driven by it. The significance of enhanced Myeov expression in gastric cancer was then investigated. As GA cells are characterised by their increased proliferative and invasive capabilities, these two functional end points were monitored *in vitro*. Using two duplexes, RNAi-mediated knockdown of Myeov in human gastric cancer cells resulted in 68 and 36% decreased proliferation in comparison with control cells in which Myeov expression was unaltered (Figure 5B). The role of Myeov in the invasivness of gastric cancers was highlighted by the decreased *in vitro* invasion of gastric cancer cells in which Myeov expression had been suppressed using siRNA (Figure 5C). Using two siRNA duplexes that targeted Myeov mRNA, cell invasion was decreased by 85 and 90% in comparison with control cells.

As Myeov is co-expressed with Cyclin D1, we propose that both genes are co-regulated and may share activity in advancing the neoplastic process. Cyclin D1 upregulation has been implicated as a driver of gastric cancer, and its inhibition has been shown to reverse the transformed phenotype of human gastric cancer cells (Chen *et al*, 1999). The degree of cyclin D1 overexpression has been correlated with invasive stages of gastric cancer (Oda *et al*, 1999).

In this study we have employed comparison of EST libraries to identify genes whose expression is putatively altered in GA. DDD. Net1 and Myeov expression was confirmed in gastric cancer tissue and cell lines. siRNA-mediated knockdown of both genes resulted in significantly decreased cell proliferation and invasion. The data presented herein suggest regulatory roles for the *in silico* identified genes Net1 and Myeov in the setting of gastric cancer. The functional consequences of knockdown of the genes provide important clues to the activity of Net1 and Myeov in GA and further study may establish the roles for these genes in initiation and progression of this disease. Further investigations will doubtlessly lend more weight to the potential of these genes as therapeutic targets in GA.

## Figures and Tables

**Table 1:** Matched gastric adenocarcinoma and normal tissue specimens used in this study

**Sample**	**Gender**	**Age (years)**	**Histology**	**Clinical stage**
1	Male	57	Adenocarcinoma	Ia
2	Male	63	Poorly differentiated adenocarcinoma	Not available
3	Female	69	Adenocarcinoma	Not available
4	Male	76	Moderately differentiated adenocarcinoma	IV
5	Male	70	Poorly differentiated adenocarcinoma	Not available
6	Female	73	Moderately differentiated adenocarcinoma	IV
7	Male	71	Poorly differentiated adenocarcinoma	Not available

**Table 2:** Details of pooled gastric cancer tissue EST libraries studied

**dbEST library ID**	**Title**	**Tissue**	**Protocol**	**Histology**	**Tumour site**	**Cell morphology**
10299	S4SNU1	Cell line	Uncharacterised	Adenocarcinoma, poorly differentiated	Primary	Lympoblast-like
10301	S5SNU484	Cell line	Uncharacterised	Adenocarcinoma, poorly differentiated	Primary	Epithelial
10302	S5SNU484s1	Cell line	Subtracted	Adenocarcinoma, poorly differentiated	Primary	Epithelial
10305	S7SNU719	Cell line	Uncharacterised	Adenocarcinoma, moderately differentiated	Primary	Epithelial
10306	S7SNU719s1	Cell line	Subtracted	Adenocarcinoma, moderately differentiated	Primary	Epithelial
10310	S10SNU1	Cell line	Uncharacterised	Adenocarcinoma, poorly differentiated	Primary	Lympoblast-like
10311	S11SNU1	Cell line	Uncharacterised	Adenocarcinoma, poorly differentiated	Primary	Lympoblast-like
10324	S21SNU520	Cell line	Uncharacterised	Adenocarcinoma, poorly differentiated	Primary	Floating aggregates
10325	S21SNU520s1	Cell line	Subtracted	Adenocarcinoma, poorly differentiated	Primary	Floating aggregates
1449	NCI_CGAP_Gas4	Bulk	Non-normalised	Adenocarcinoma, poorly differentiated	Primary	Bulk tissue
3637	ST0240	Bulk	Uncharacterised	Carcinoma	Primary	Bulk tissue

**Table 3:** Novel genes identified by DDD

**Symbol**	**Name**	**UniGene ID**	**GA^a^**	**Non cancer^a^**	**Cancer^a^**	**Function**
AGR2	Arginase, type II	Hs.226391	0.00242	0.00012	0.00017	Urea cycle
ANKRD9	Ankyrin repeat domain 9	Hs.432945	0.00098	0.00002	0.00004	Function unknown
ARHGEF16	Rho guanine exchange factor 16	Hs.87435	0.00032	0.00002	0.00002	Function unknown
BENE	BENE protein	Hs.185055	0.00059	0.00003	0.00004	Function unknown
C8G	Complement component 8, gamma polypeptide	Hs.1285	0.00016	0.00000	0.00000	Complement activation
CLDN18	Claudin 18	Hs.278966	0.00043	0.00003	0.00001	Tight junction component
CLDN2	Claudin 2	Hs.16098	0.00023	0.00002	0.00002	Tight junction component
EPS8L3	EPS8-like 3	Hs.5366	0.00021	0.00001	0.00002	Receptor activity
FBXL6	F-box and leucine-rich repeat protein 6	Hs.12271	0.00037	0.00002	0.00003	Ubiquitin cycle
GEFT	RAC/CDC42 exchange factor	Hs.61581	0.00027	0.00002	0.00003	Cell proliferation
KIAA1706	KIAA1706 protein	Hs.412318	0.00034	0.00003	0.00001	DNA binding
KREMEN2	Kringle containing transmembrane protein 2	Hs.73452	0.00043	0.00000	0.00004	Wnt signaling pathway
MEIS4	Likely ortholog of mouse myeloid ecotropic viral integration site-related gene 2	Hs.356135	0.00043	0.00002	0.00004	Transcription regulation
***MYEOV***	***Myeloma overexpressed gene***	***Hs.436000***	***0.00048***	***0.00000***	***0.00004***	***Nucleic acid binding***
***NET1***	***Neuroepithelial cell transforming gene 1***	***Hs.25155***	***0.00100***	***0.00008***	***0.00008***	***Regulation of cell growth***
NOS3	Nitric-oxide synthase activity	Hs.511603	0.00027	0.00002	0.00001	Nitric-oxide synthase activity
PARD6G	Par-6 partitioning defective 6 homolog gamma (*Caenorhabditis elegans*)	Hs.223584	0.00037	0.00001	0.00001	Cytokinesis
PTK2B	Protein tyrosine kinase 2 beta	Hs.438975	0.00094	0.00004	0.00005	Signal transduction
S100A14	S100 calcium binding protein A14	Hs.288998	0.00041	0.00001	0.00003	Calcium ion binding
SMCX	Smcx homolog, X chromosome (mouse)	Hs.103381	0.00066	0.00004	0.00004	Transcription regulation
THBS3	Thrombospondin 3	Hs.169875	0.00046	0.00004	0.00003	Cell-matrix adhesion
UNC93A	Unc-93 homolog A (*C. elegans*)	Hs.267749	0.00027	0.00000	0.00000	Function unknown
WBSCR21	Williams beuren syndrome chromosome region 21	Hs.182476	0.00043	0.00002	0.00004	Aromatic compound metabolism

a Fraction of sequences mapping to UniGene cluster.
